# MICU1-dependent mitochondrial calcium uptake regulates lung alveolar type 2 cell plasticity and lung regeneration

**DOI:** 10.1172/jci.insight.154447

**Published:** 2022-02-22

**Authors:** Mir Ali, Xiaoying Zhang, Ryan LaCanna, Dhanendra Tomar, John W. Elrod, Ying Tian

**Affiliations:** Department of Cardiovascular Sciences, Center for Translational Medicine, Lewis Katz School of Medicine at Temple University, Philadelphia, Pennsylvania, USA

**Keywords:** Cell Biology, Stem cells, Bacterial infections, Calcium, Mitochondria

## Abstract

Lung alveolar type 2 (AT2) cells are progenitors for alveolar type 1 (AT1) cells. Although many factors regulate AT2 cell plasticity, the role of mitochondrial calcium (_m_Ca^2+^) uptake in controlling AT2 cells remains unclear. We previously identified that the miR-302 family supports lung epithelial progenitor cell proliferation and less differentiated phenotypes during development. Here, we report that a sustained elevation of miR-302 in adult AT2 cells decreases AT2-to-AT1 cell differentiation during the *Streptococcus pneumoniae*–induced lung injury repair. We identified that miR-302 targets and represses the expression of mitochondrial Ca^2+^ uptake 1 (MICU1), which regulates _m_Ca^2+^ uptake through the _m_Ca^2+^ uniporter channel by acting as a gatekeeper at low cytosolic Ca^2+^ levels. Our results reveal a marked increase in MICU1 protein expression and decreased _m_Ca^2+^ uptake during AT2-to-AT1 cell differentiation in the adult lung. Deletion of *Micu1* in AT2 cells reduces AT2-to-AT1 cell differentiation during steady-state tissue maintenance and alveolar epithelial regeneration after bacterial pneumonia. These studies indicate that _m_Ca^2+^ uptake is extensively modulated during AT2-to-AT1 cell differentiation and that MICU1-dependent _m_Ca^2+^ uniporter channel gating is a prominent mechanism modulating AT2-to-AT1 cell differentiation.

## Introduction

The mammalian lung alveolar epithelium comprises two main cell types: gas-exchanging squamous alveolar type 1 (AT1) cells and cuboidal alveolar type 2 (AT2) cells. AT2 cells synthesize and secrete surfactants, regulate the innate immune response, and act as progenitors for AT1 cells (1–4). Models of lung damage in mice have demonstrated that AT2 cells increase their proliferation and differentiation into AT1 cells in response to injury. These AT2 cell activities are vital to alveolar epithelial repair and regeneration (1, 2). Because AT2 cells are the key player of alveolar epithelial repair and regeneration after injury, it is important to define the molecular mechanisms that mediate their function.

Calcium homeostasis is fundamental for cell proliferation, differentiation, cell death, and inflammation. Mitochondria are important components of calcium signaling because they modulate both the amplitude and the spatial-temporal patterns of cytosolic calcium (_c_Ca^2+^) signals and cellular energetics (5–7). Elevation in _c_Ca^2+^ level is rapidly integrated into mitochondria through the mitochondrial calcium uniporter channel complex (mtCU), because of the high electromotive force generated by the electron transport chain (5, 6). Mitochondrial calcium (_m_Ca^2+^) regulates bioenergetics by activating dehydrogenases in the Krebs cycle and modulating components of the electron transport chain (8–10). AT2 cells display a much greater volume density of mitochondria than other lung cells, such as endothelial cells or AT1 cells (11, 12), suggesting a pivotal role for _m_Ca^2+^ signaling. However, the role of _m_Ca^2+^ uptake in AT2 cell function remains elusive, in part because the molecular identity of the mtCU has been only recently determined (7, 13–17)

The mtCU comprises many components, including the mitochondrial calcium uniporter (MCU), which is the pore-forming component and necessary for Ca^2+^ permeation of the inner mitochondrial membrane (5–7, 15). Whole-body MCU KO mice were embryonic lethal on a C57BL/6 background (18, 19), suggesting a central role for _m_Ca^2+^ uptake in development. MCU-mediated Ca^2+^ flux is regulated by mitochondrial calcium uptake 1 (MICU1), which keeps the MCU in a closed conformation at low _c_Ca^2+^ levels and thereby regulates mitochondrial signaling and function (13, 14, 20, 21). A loss-of-function mutation of MICU1 has been linked to human disease through alterations in _m_Ca^2+^ handling (22–24), and germline deletion of MICU1 is perinatally lethal in mice (25). MICU1 deletion in adult tissues led to decreased liver regeneration and skeletal muscle repair in mice (25, 26). MICU1 regulates some cellular differentiation programs via its role in modulating matrix Ca^2+^ content, energetics, and metabolite bioavailability for the activation of chromatin-remodeling enzymes to modulate gene expression (27, 28).

Despite these important findings, the role of _m_Ca^2+^ uptake in regulating AT2 cell plasticity and function remains unknown. In our previous studies, we identified that the miRNA family miR-302 supports cell proliferation, but less-differentiated phenotypes, in cardiomyocytes and lung epithelial progenitor cells during development (29, 30). miR-302 regulates cell proliferation, in part, through repression of the Hippo signaling pathway and promotion of Yap/Taz nuclear activities (30). In a previous model of alveolar epithelial regeneration during bacterial pneumonia, we demonstrated that Yap/Taz nuclear activities are required for AT2 cell differentiation into AT1 cells (31). This prompted us to explore whether miR-302 promotes the differentiation of AT2 cells into AT1 cells in adult lungs. To our surprise, a sustained elevation of miR-302 level in AT2 cells reduced the differentiation of AT2 cells into AT1 cells even in Yap/Taz KO mice, indicating the decreased differentiation capacity mediated by miR-302 was independent of Hippo signaling. We identified that miR-302 targets and represses the expression of the mtCU channel regulator, MICU1. Analysis of MICU1 expression and _m_Ca^2+^ transient in adult lungs revealed a marked increase in MICU1 protein and decreased _m_Ca^2+^ uptake during AT2 cell differentiation into AT1 cells. Deletion of *Micu1* in AT2 cells reduced AT2 cell differentiation into AT1 cells during steady-state tissue maintenance and alveolar epithelial regeneration after bacterial pneumonia. The results of these studies indicate that _m_Ca^2+^ uptake is extensively modulated during AT2 cell differentiation into AT1 cells and that _m_Ca^2+^ uptake, via MICU1-dependent mtCU gating, plays a critical role in regulating AT2 cell differentiation into AT1 cells during lung homeostasis and alveolar epithelial regeneration.

## Results

### Sustained elevation of miR-302 in AT2 cells decreases the differentiation of AT2 cells into AT1 cells.

**Figure 1:**
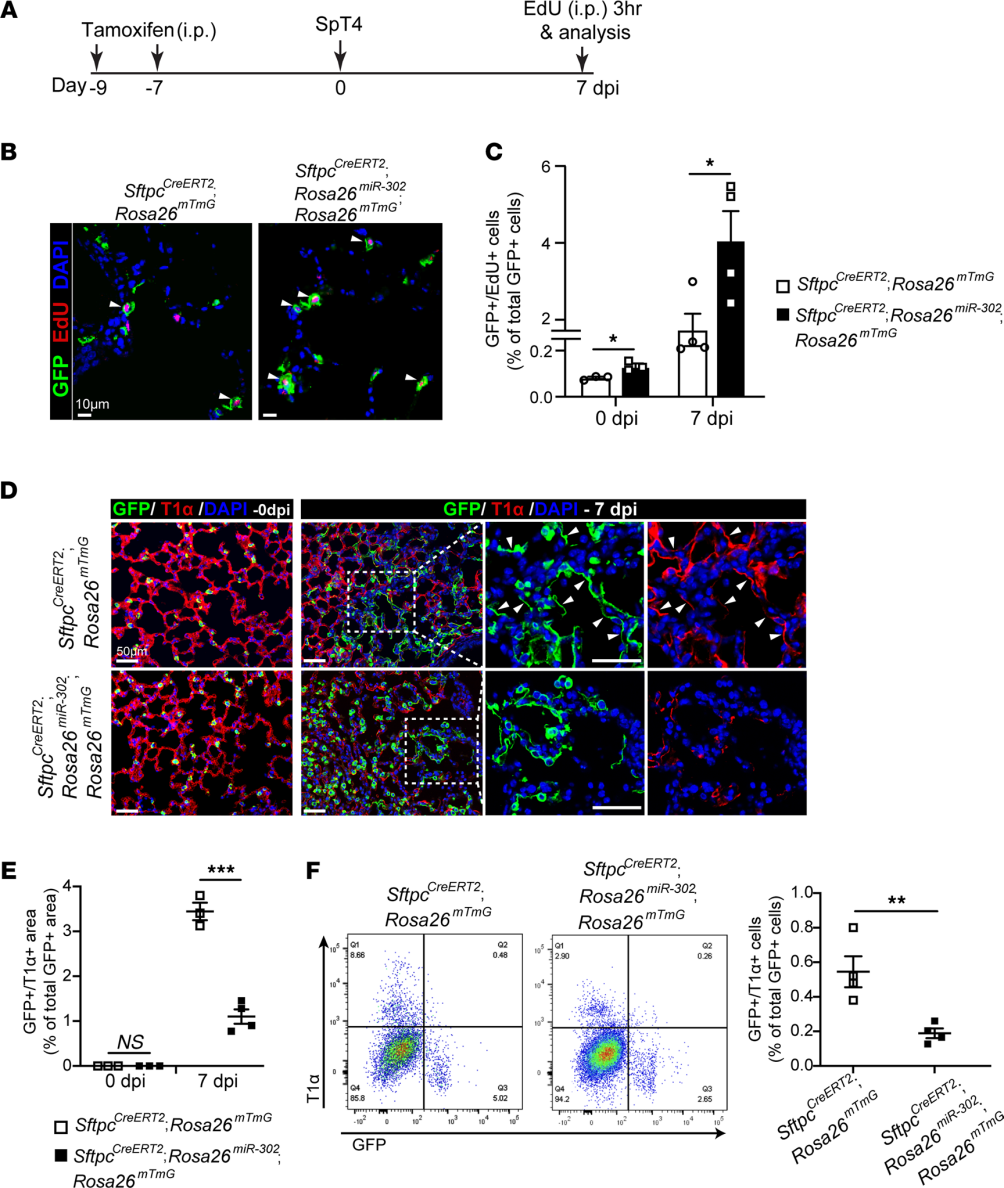
Sustained elevation of miR-302 in AT2 cells reduces AT2 cell differentiation into AT1 cells. (**A**) Adult *Sftpc^CreERT2^*; *Rosa26^mTmG^* or *Sftpc^CreERT2^*; *Rosa26^miR-302^*; *Rosa26^mTmG^* mice received two doses of tamoxifen to label *Sftpc*^+^ AT2 cells. Mice were infected with SpT4 after 7 days from last tamoxifen treatment and were examined at 7 dpi. (**B**) Confocal images of lung sections at 7 dpi. Lineage-labeled AT2 cells in the DNA synthesis phase were detected using Click-iT EdU Alexa Fluor (red) and co-immunostaining with antibody against GFP (green) to detect GFP^+^ cells. The cell nucleus was stained with DAPI (blue). Arrows point to regions double positive for GFP and EdU. Scale bar: 10 μm. (**C**) Quantification of EdU^+^/GFP^+^ cells as a percentage of total GFP^+^ cells analyzed (~2200 GFP^+^ cells per animal). (**D**) Immunostaining of lung sections with antibodies to GFP (green) and T1α (red), an AT1 cell marker, to detect the differentiation of lineage-labeled AT2 cells into AT1 cells. Arrows point to regions double positive for GFP and T1α. Scale bar: 50 μm. (**E**) Quantification of the percentage of GFP^+^/T1α^+^ area of total GFP^+^ area per field using ImageJ software. (**F**) Flow cytometry analysis of dissociated lung cells. The percentage of GFP^+^/T1α^+^ cells of total GFP^+^ cells at 7 dpi is shown. Data are presented as mean ± SEM. *P* values were calculated using Student’s *t* test. *P < 0.05; **P < 0.01; ***P < 0.001.

miR-302 is expressed in embryonic lungs but declines rapidly after embryonic day 14.5 and is undetectable in the postnatal lung (29). We generated *Sftpc^CreERT2^*; *Rosa26^miR-302^*; *Rosa26^mTmG^* mice to specifically overexpress miR-302 and label AT2 cells in the adult lung (Supplemental Figure 1A; supplemental material available online with this article; https://doi.org/10.1172/jci.insight.154447DS1). We confirmed high expression levels of all members of the miR-302 cluster in purified AT2 cells after tamoxifen administration by quantitative real-time PCR (qPCR; Supplemental Figure 1, B and C). The number of lineage-labeled (GFP^+^) AT2 cells re-entering the cell cycle was significantly increased in *Sftpc^CreERT2^*; *Rosa26^miR-302^*; *Rosa26^mTmG^* lungs, as compared with *Sftpc^CreERT2^*; *Rosa26^mTmG^* control lungs both before (0 days after infection [dpi]) and 7 dpi infection with *Streptococcus pneumoniae* strain T4 (SpT4; Figure 1, A–C). This result is consistent with our previous findings that overexpression of miR-302 promotes cell proliferation (29, 30, 32). TUNEL staining of lung sections showed no significant difference in cell death between *Sftpc^CreERT2^*; *Rosa26^miR-302^* lungs and *Sftpc^CreERT2^* lungs at 7 dpi (Supplemental Figure 1, D and E). We examined the differentiation of AT2 cells into AT1 cells by quantifying the percentage of GFP^+^ alveolar surface area covered by AT2-derived AT1 cells (GFP^+^/T1α^+^) on sectioned lungs. We observed a significant decrease in the level of AT2 cell differentiation into AT1 (GFP^+^/T1α^+^) cells in *Sftpc^CreERT2^*; *Rosa26^miR-302^*; *Rosa26^mTmG^* lungs compared with control lungs at 7dpi (Figure 1, D and E). Consistent with this finding, FACS analysis showed a significant reduction in the level of AT2 cell differentiation into AT1 (GFP^+^/T1α^+^) cells in *Sftpc^CreERT2^*; *Rosa26^miR-302^*; *Rosa26^mTmG^* lungs (Figure 1F).

### miR-302–dependent loss of AT2 to AT1 cell differentiation is independent of Yap/Taz (Hippo) signaling.

Our previous studies demonstrated that miR-302 functions, in part, by inhibiting several components of the Hippo signaling pathway and promoting Yap/Taz nuclear activities (30). To determine whether the reduced differentiation of AT2 cells to AT1 cells in *Sftpc^CreERT2^*; *Rosa26^miR-302^* lungs was due to the inhibition of Hippo signaling, we deleted Yap/Taz expression in miR-302–overexpressed AT2 cells using *Sftpc^CreERT2^*; *Rosa26^miR-302^*; *Yap^fl/fl^*; *Taz^fl/fl^*; *Rosa26^mTmG^* mice (Supplemental Figure 2A). If Hippo signaling inhibition was responsible for the decreased differentiation of AT2 cells into AT1 cells, *Sftpc^CreERT2^*; *Rosa26^miR-302^*; *Yap^fl/fl^*; *Taz^fl/fl^*; *Rosa26^mTmG^* mice should have improved AT2-to-AT1 cell differentiation compared with *Sftpc^CreERT2^*; *Rosa26^miR-302^*; *Rosa26^mTmG^* mice. However, we observed similar percentages of AT1 cells derived from preexisting AT2 (GFP^+^/T1α^+^) cells in both groups (Supplemental Figure 2, B and C). These findings indicate that the reduced AT2-to-AT1 cell differentiation in *Sftpc^CreERT2^*; *Rosa26^miR-302^*; *Rosa26^mTmG^* lungs is independent of the miR-302–Hippo signaling axis.

### miR-302 represses MICU1 expression and induces changes in AT2 cell mitochondrial structure.

**Figure 2:**
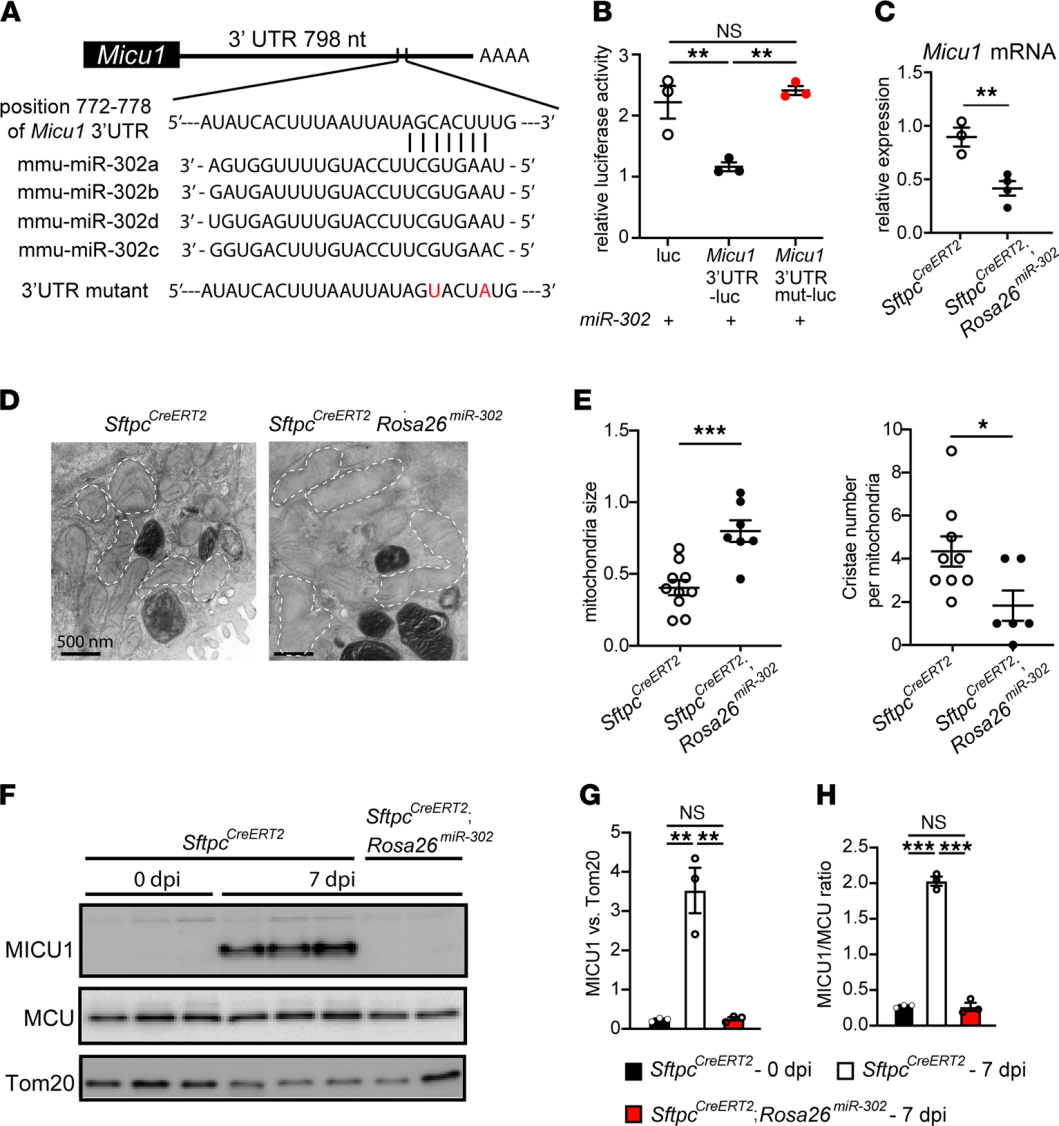
miR-302 targets and represses MICU1 expression. (**A**) Predicted binding site of miR-302 on 3′-UTR of *Micu1*. (**B**) HEK293T cells were transfected with both the empty LUC reporter or micu1 3′-UTR reporter (*Micu1* 3′-UTR-luc) or micu1 3′-UTR reporter with mutation of the miR-302 binding site (*Micu1* 3′-UTR mut-luc) and an expression plasmid for miR-302. Cell extracts were assayed for LUC expression at 48 hours after transfection. LUC reporter assays showing that miR-302 can repress *Micu1* expression through its 3′-UTR. This repression can be reversed by mutation of the miR-302 binding site. (**C**) Adult *Sftpc^CreERT2^* or *Sftpc^CreERT2^*; *Rosa26^miR-302^* mice received two doses of tamoxifen. AT2 cells were purified after 7 days from last tamoxifen treatment, and expression of *Micu1* was examined by qPCR. Gene expression was normalized to *Rps13*, a mitochondrial gene. (**D**) Transmission electron microscopy of lungs 3 weeks after tamoxifen treatment showed disruptive mitochondrial morphology and cristae structure in *Sftpc^CreERT2^*; *Rosa26^miR-302^* AT2 cells. Dashed white lines outline mitochondria. (**E**) Quantification of mitochondria size and cristae numbers per mitochondrion, using ImageJ. (**F**) Expression of MICU1 and MCU proteins in mouse lungs as analyzed by Western blot. Tom20 was the mitochondrial loading control. (**G**) Densitometry chart showing the relative protein level of MICU1. Band density was normalized to Tom20. (**H**) Graph of the ratio of MICU1 to MCU determined by Western blot. Data are presented as mean ± SEM. *P* values were calculated using 1-way ANOVA (**B**, **G**, and **H**) and Student’s *t* test (**C** and **E**).*P < 0.05; **P < 0.01; ***P < 0.001.

Using databases of TargetScan and miRWalk (33, 34), there is a predicted interaction between miR-302 and *Micu1*, suggesting miR-302 is a negative regulator of MICU1 expression (Figure 2A). We validated that miR-302 repressed *Micu1* expression through its 3′-UTR (Figure 2B). Overexpression of miR-302 in AT2 cells from *Sftpc^CreERT2^*; *Rosa26^miR-302^* mouse lungs led to decreased expression of *Micu1* (Figure 2C). Ultrastructural examination of *Sftpc^CreERT2^*; *Rosa26^miR-302^* lungs at 3 weeks after tamoxifen treatment revealed disrupted mitochondrial morphology and cristae structure in AT2 cells (Figure 2D). Quantitatively, AT2 cells from *Sftpc^CreERT2^*; *Rosa26^miR-302^* lungs exhibited increased mitochondrial area and decreased cristae density per mitochondrion, compared with *Sftpc^CreERT2^* controls (Figure 2E). These results suggest that decreased AT2-to-AT1 cell differentiation in *Sftpc^CreERT2^*; *Rosa26^miR-302^* lungs may be due to miR-302 inhibiting MICU1 expression, thereby affecting mitochondrial structure and function.

### Lung injury induces MICU1 expression in AT2 cells during AT2-to-AT1 cell differentiation.

Our previous microarray analysis on lineage-labeled AT2 cells isolated from *Sftpc^CreERT2^*; *Rosa26^mTmG^* mouse lungs showed high expression of genes associated with chromatin organization, histone modification, TCA cycle, mitochondrial inner membrane, and a 2.3-fold increase in *Micu1* expression at day 8 after SpT4 infection, the period when AT2 cells differentiate into AT1 cells (Supplemental Figure 2D) (31). qPCR analysis confirmed increased *Micu1* expression in AT2 cells of SpT4-infected mice at 7 dpi compared with uninfected mice (0 dpi) (Supplemental Figure 2E). The expression of other components of the mtCU, including *Mcu, Emre*, and *Mcur1*, was unaffected (Supplemental Figure 2E). Furthermore, Western blot analysis revealed a marked increase in MICU1 protein level in mouse lungs at 7 dpi (Figure 2, F and G). Tom20 served as a mitochondrial loading control. Because the MICU1-to-MCU ratio underlies tissue-specific differences in the mtCU _m_Ca^2+^ threshold of uptake (35), we quantified the relative change in MICU1/MCU expression. Lungs at 7 dpi showed a rapid increase in the MICU1-to-MCU ratio (Figure 2H). In contrast, MICU1 protein production was dramatically reduced in *Sftpc^CreERT2^*; *Rosa26^miR-302^* lungs (Figure 2, F–H), which is consistent with the finding that miR-302 targets and represses MICU1 expression. Together, these data suggest a potential role for _m_Ca^2+^ uptake, via MICU1-dependent mtCU gating, in regulating AT2-to-AT1 cell differentiation after SpT4-induced lung injury.

### AT2-to-AT1 cell differentiation correlates with changes in mtCU gating and reduced _m_Ca^2+^ uptake.

**Figure 3:**
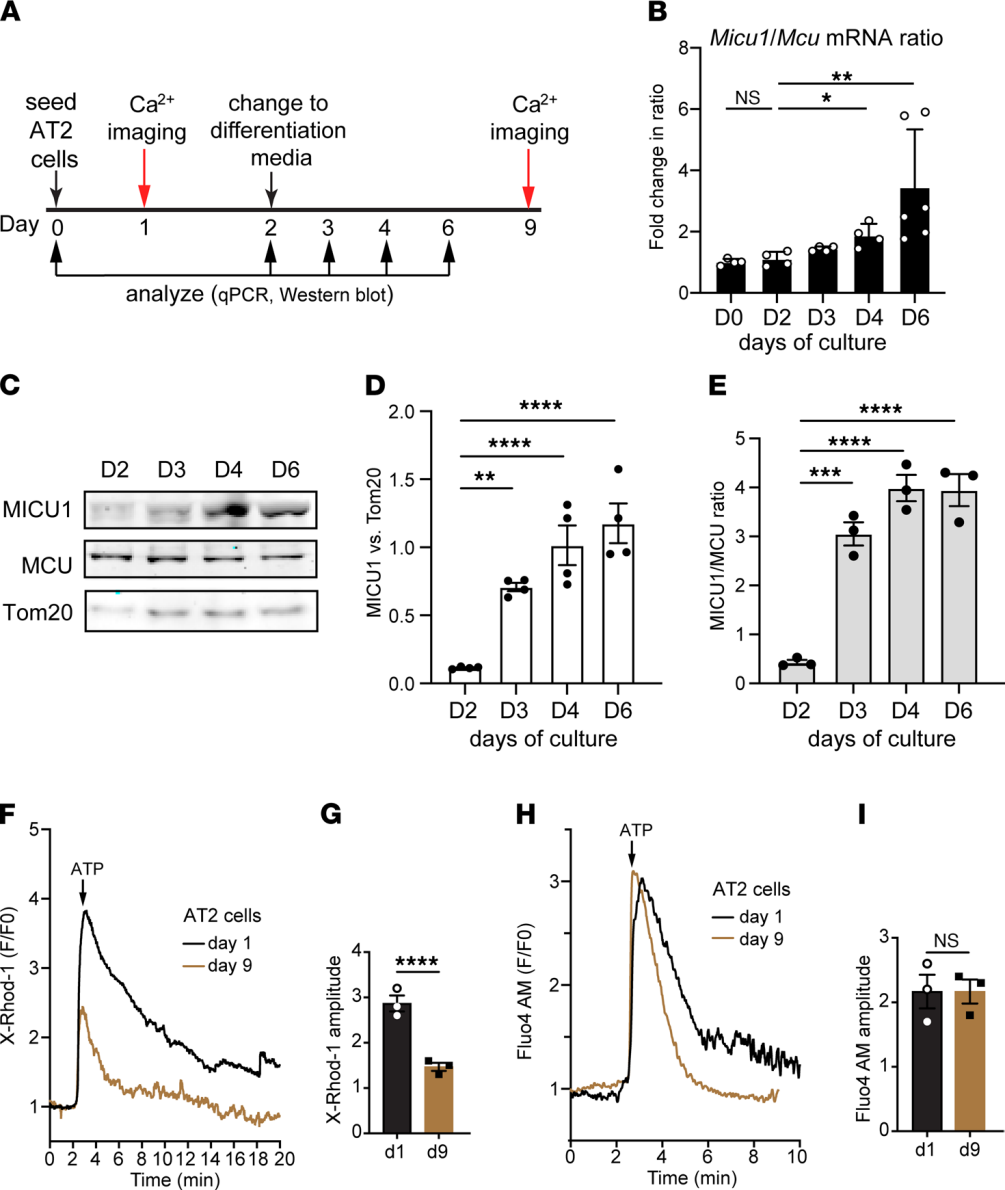
Increased MICU1 expression and decreased _m_Ca^2+^ uptake are associated with AT2 cell differentiation into AT1 cells. (**A**) Schematic of experimental design showing the time line of cell differentiation and analysis of AT2 cells from WT adult mice. (**B**) The fold change of *Micu1* to *Mcu* mRNA ratio by qPCR from cells at day (D) 2, D3, D4, and D6 of culture. (**C**) Western blots of whole-cell protein showing the expression of MICU1 and MCU. Tom20 was the mitochondrial loading control. (**D**) Densitometry chart showing the relative protein level of MICU1. Band density was normalized to Tom20. (**E**) Graph of the ratio of MICU1 to MCU by Western blot. (**F**) Measurement of _m_Ca^2+^ uptake in undifferentiated AT2 cells (day 1) and differentiated AT2 cells (day 9) as assessed by the _m_Ca^2+^ sensor, X-Rhod-1. To initiate IP3R-mediated Ca^2+^ release, 1 mM ATP was delivered. (**G**) Graph of amplitude (peak intensity) of X-Rhod-1. (**H**) Measurement of _c_Ca^2+^ uptake in undifferentiated and differentiated AT2 cells as assessed by the _c_Ca^2+^-sensitive dye Fluo4 AM. (**I**) Graph of amplitude (peak intensity) of Fluo4 AM. Data are presented as mean ± SEM. *P* values were calculated using 1-way ANOVA (**B**, **D**, and **E**) and Student’s *t* test (**G** and **I**).*P < 0.05; **P < 0.01; ***P < 0.001; ****P < 0.0001.

To assess the impact of _m_Ca^2+^ uptake on AT2-to-AT1 cell differentiation, we examined mtCU gating and _m_Ca^2+^ dynamics using the in vitro 2D culture model in which primary mouse AT2 cells transdifferentiate into AT1-like cells within 7 days of culture (36, 37). First, we evaluated the expression profiles of MICU1 and MCU in mouse AT2 cells at different stages of differentiation and observed an increase in the MICU1-to-MCU ratio from day 2 to day 6 of culture (Figure 3, A–E). qPCR analysis revealed a significant increase in the *Micu1* to *Mcu* mRNA ratio in AT2 cells at 4 days of culture and onward (Figure 3B). The increase in the *Micu1* to *Mcu* mRNA ratio was consistent with the progressive increase in MICU1 to MCU protein ratio in differentiated AT2 cells (Figure 3, C–E).

Next, we measured _m_Ca^2+^ uptake in AT2 cells by live-cell imaging. AT2 cells purified from WT adult mice were seeded on rat-tail collagen–coated plates and, 24 hours later, were loaded with MitoTracker Green and X-Rhod-1, a Ca^2+^-sensitive dye that accumulates in mitochondria (Figure 3A) (38, 39). We first confirmed that X-Rhod-1 colocalized to the mitochondria and cell permeabilization with digitonin did not change the mitochondrial signals (Supplemental Figure 2, F–I). These data indicate that X-Rhod-1 is, indeed, measuring _m_Ca^2+^ and not Ca^2+^ from other subcellular sources. In this assay, AT2 cells (day 1 of culture) challenged with ATP, purinergic inositol 1,4,5-triphosphate receptor (IP3R) calcium stimulus, displayed a robust increase in _m_Ca^2+^ levels (Figure 3, F and G). AT2 cells at 7 days after differentiation (day 9 of culture) showed a significant decrease in total _m_Ca^2+^ uptake after the ATP stimulus (Figure 3, F and G). No significant differences in _c_Ca^2+^ transients were observed between undifferentiated (day 1 of culture) and differentiated (day 9 of culture) AT2 cells, as revealed by the cytosolic Ca^2+^-sensitive dye Fluo-4 AM (Figure 3, H and I). These results indicate that _m_Ca^2+^ uptake is extensively modulated during AT2 cell differentiation into AT1 cells.

### MICU1 deletion in AT2 cells decreases AT2-to-AT1 cell differentiation both in vitro and in vivo.

**Figure 4:**
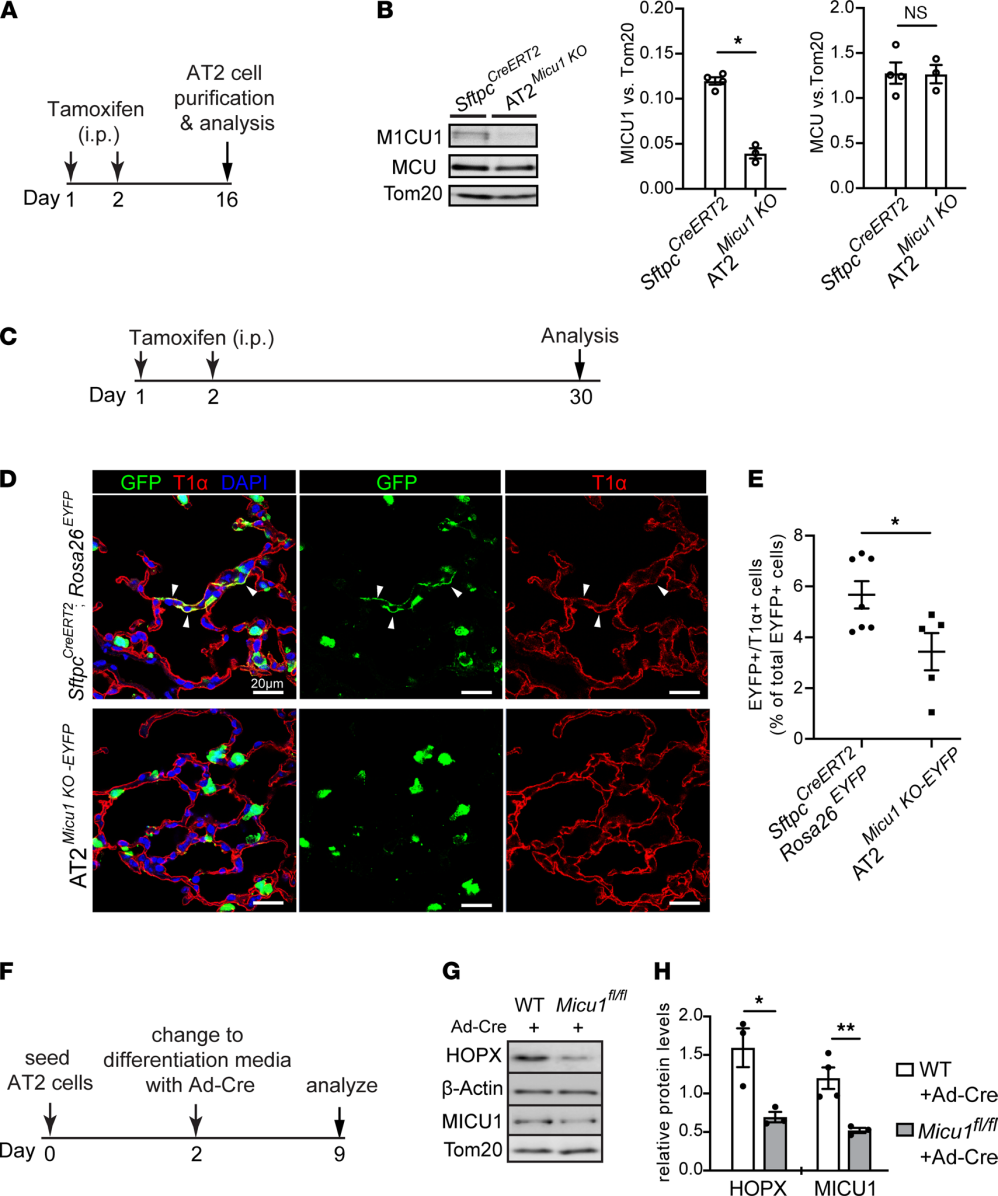
MICU1 expression is necessary for AT2 cell differentiation into AT1 cells during homeostatic tissue maintenance. (**A**) Schematic of experimental design for studies performed in **B** showing time line of tamoxifen treatment and AT2 cell harvest from adult mice. (**B**) Western blots of whole-cell protein from purified AT2 cells from *Sftpc^CreERT2^* and *Sftpc^CreERT2^*; *Micu1^fl/fl^* (AT2*^Micu1^^KO^*) mice. The graphs of fold change in the protein levels of MICU1 and MCU by Western blot are shown on the right. Band density was normalized to Tom20. (**C**) Schematic of experimental design for studies performed in **D** and **E**. (**D**) Confocal images of lung sections at 4 weeks after last dose of tamoxifen treatment, with antibodies to GFP (green) and T1α (red). GFP antibody was used to detect EYFP^+^ cells. The cell nucleus was stained with DAPI (blue). Arrows point to regions double positive for GFP and T1α. Scale bars: 20 μm. (**E**) Flow cytometry analysis of dissociated lung cells showing the percentage of EYFP^+^/T1α^+^ cells of total EYFP^+^ cells at 4 weeks after last dose of tamoxifen treatment. (**F**) Schematic of experimental design for studies performed in **G** and **H**. Purified AT2 cells from adult WT or *Micu1^fl/fl^* mouse lungs were seeded on rat-tail collagen–coated plates. At day 2 of culture, AT2 cells were infected with Ad-Cre and treated with differentiation medium; they were examined at 7 days after differentiation (day 9 of culture). (**G**) Western blots of whole-cell protein from control (WT+Ad-Cre) and *Micu1*-deleted AT2 (*Micu1^fl/fl^*+Ad-Cre) cells at day 9 of culture showing the expression of HOPX (an AT1 cell marker) and MICU1.Tom20 and β-actin were the mitochondrial loading control and total lysate loading control, respectively. (**H**) Densitometry chart showing relative protein levels of HOPX and MICU1. Band densities of HOPX and MICU1 were normalized to β-actin and Tom20, respectively. Data are presented as mean ± SEM. *P* values were calculated using Student’s *t* test. *P < 0.05; **P < 0.01.

To test if _m_Ca^2+^ uptake, via MICU1-dependent mtCU gating, is required for the differentiation of AT2 cells into AT1 cells, *Micu1^fl/fl^* mice were crossed with the well-characterized *Sftpc^CreERT2^* knock-in mouse model to yield AT2-specific loss of *Micu1*. *Micu1* deletion was induced in *Sftpc^CreERT2^*; *Micu1^fl/fl^* (AT2*^Micu1^^KO^*) adult mice by i.p. administration of tamoxifen followed by an additional 2-week period to allow for MICU1 protein turnover and tamoxifen washout (Figure 4A). Western blot analyses showed an approximately 75% reduction in MICU1 protein expression in purified AT2 cells of AT2*^Micu1^^KO^* mice, compared with *Sftpc^CreERT2^* age-matched controls (Figure 4B). No significant changes in the expression of other mtCU regulatory subunits, including MCU, MICU2, and MCU regulator 1 (MCUR1), were found in AT2*^Micu1^^KO^* AT2 cells (Figure 4B and Supplemental Figure 3A).

To determine the impact of *Micu1* deletion on _m_Ca^2+^ dynamics, AT2*^Micu1^^KO^* and control AT2 primary cells were purified and imaged after 24 hours of culture (Supplemental Figure 3B). AT2*^Micu1^^KO^* AT2 cells had higher levels of _m_Ca^2+^ uptake and lower levels of _c_Ca^2+^ load than did *SftpcCreERT2* control AT2 cells (Supplemental Figure 3, C–F). Because AT2 cells rely on Ca^2+^ signaling to generate ATP needed to produce pulmonary surfactants (11, 40, 41), we investigated if loss of MICU1 altered lung surfactant production. Western blots of lung tissues showed similar levels of surfactant proteins such as SP-A, SP-B, and SP-D in AT2*^Micu1^^KO^* and *SftpcCreERT2* control lungs (Supplemental Figure 3G), indicating that the surfactant protein synthesis was not affected in AT2*^Micu1^^KO^* lungs. We also evaluated the level of phosphatidylcholine, the main component of surfactant lipid, in bronchoalveolar lavage fluid (BALF) using a phosphatidylcholine colorimetric assay. No significant differences in phosphatidylcholine content were observed among AT2*^Micu1^^KO^*, *SftpcCreERT2* control, and WT lungs (Supplemental Figure 3H). These results indicate that loss of *Micu1* in AT2 cells does not affect surfactant biosynthesis.

Next, by IHC and flow cytometry analysis, we examined and quantified the differentiation of lineage-labeled *Sftpc*^+^ AT2 cells in *Sftpc^CreERT2^*; *Micu1^fl/fl^*; *Rosa26^EYFP^* (AT2*^Micu1^^KO-EYFP^*) mice 4 weeks after tamoxifen administration (Figure 4, C–E). AT2*^Micu1^^KO-EYFP^* mice exhibited a reduced percentage of lineage-labeled AT2 cells that had differentiated into AT1 cells (EYFP^+^/T1α^+^), as compared with *Sftpc^CreERT2^*; *Rosa26^EYFP^* controls (Figure 4, D and E). To determine the contribution of _m_Ca^2+^ uptake to the differentiation of AT2 cells into AT1 cells in in vitro culture, AT2 cells were purified from adult *Micu1^fl/fl^* or WT mouse lungs. At day 2 of culture, all groups were infected with adenovirus expression Cre recombinase (Ad-Cre) and treated with differentiation medium (serum-free medium) to induce cellular differentiation for 7 days (Figure 4F). AT2 cells were examined for differentiation into AT1 cells by quantifying the expression of homeodomain-only protein (HOPX; an AT1 cell marker) by Western blot. *Micu1*-deleted AT2 cells (*Micu1^fl/fl^*+Ad-Cre) showed reduced AT1 cell formation, as evidenced by a decrease in HOPX protein levels compared with that in control AT2 cells (WT+Ad-Cre) (Figure 4, G and H). These results indicate that loss of *Micu1* in AT2 cells inhibits AT2 cell differentiation into AT1 cells during steady-state tissue maintenance and in vitro culture.

### Loss of MICU1 in AT2 cells impairs alveolar epithelial repair and regeneration after injury.

The rapid upregulation of MICU1 protein in AT2 cells of SpT4-injured lungs, as shown in Figure 2F, suggests a potential role for MICU1 in alveolar regeneration, likely by promoting AT2-to-AT1 cell differentiation, given the aforementioned results. We tested this hypothesis using AT2*^Micu1^^KO-EYFP^* mice. Loss of MICU1 proteins were verified by Western blot analysis performed on purified AT2 cells 14 days after tamoxifen administration (Figure 4, A and B). We found no detectable changes in lineage-labeled (GFP^+^) AT2 cell DNA synthesis, cell apoptosis, and pulmonary function of forced expiratory volume in 0.05 seconds (FEV_0.05_) or forced vital capacity (FVC) as assessed by 5-ethynyl-2′-deoxyuridine (EdU) incorporation assay, TUNEL staining, and flexiVent (SciReq), respectively (Supplemental Figure 4, A–D).

**Figure 5:**
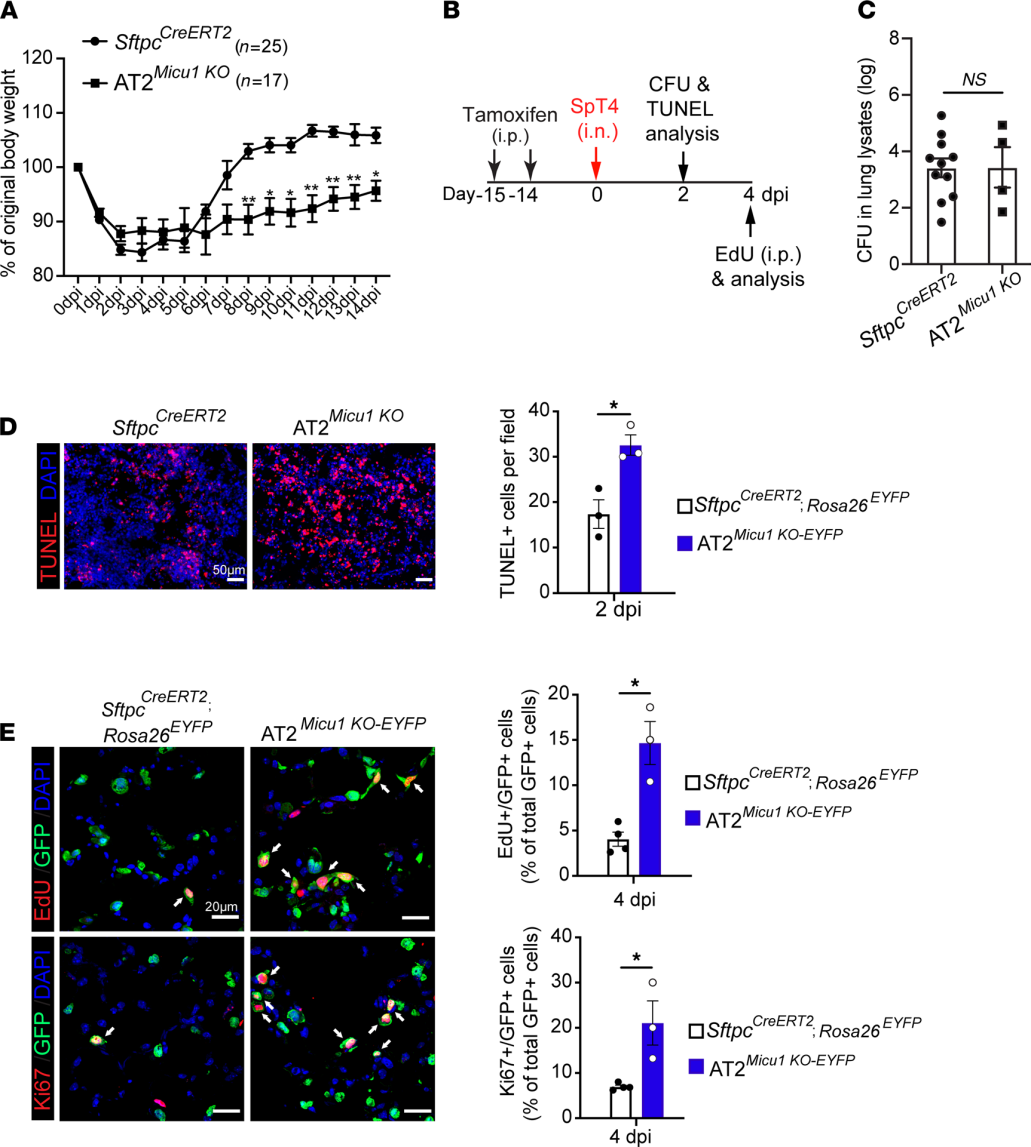
MICU1 deletion in AT2 cells impairs epithelial repair and animal recovery from bacterial pneumonia. (**A**) Measurements of body weights of adult *Sftpc^CreERT2^* and *Sftpc^CreERT2^*; *Micu1^fl/fl^* (AT2*^Micu1^^KO^*) mice. (**B**) Schematic of experimental design for studies performed in **C–E**. (**C**) Mouse lungs at 2 dpi were homogenized and lung lysates were plated for quantitative culture of colonizing pneumococci. (**D**) TUNEL staining of lung sections at 2 dpi. Graph on the right shows the quantification of cell apoptosis by counting TUNEL+ cells in lung sections. Scale bar: 50 μm. (**E**) Confocal images of lung sections at 4 dpi. Lineage-labeled AT2 cells in the cell-cycle progression were detected using Click-iT EdU Alexa Fluor (red, upper panel) or antibody against Ki67 (red, lower panel) and co-immunostaining with antibody against GFP (green). GFP antibody was used to detect EYFP+ cells. The cell nucleus was stained with DAPI (blue). Arrows point to regions double positive for GFP and EdU or Ki67. Graphs on the right show quantification of EdU+/GFP+ cells or Ki67+/GFP+ cells as a percentage of total GFP+ cells analyzed (~1000 GFP+ cells per animal). Scale bar: 20 μm. Data are presented as mean ± SEM. *P* values were calculated using 2-way ANOVA (**A**) and Student’s *t* test (**C–E**). *P < 0.05; **P < 0.01.

To determine the contribution of MICU1-dependent _m_Ca^2+^ uptake to alveolar epithelial regeneration, AT2*^Micu1^^KO^* mice were exposed to SpT4-induced lung injury. AT2*^Micu1^^KO^* mice displayed a delay in recovery from bacterial pneumonia, regaining body weight more slowly than did *Sftpc^CreERT2^* control mice (Figure 5A). We profiled lung bacterial loads by measuring CFUs at 2 dpi and found no differences in bacteria number in lung homogenates from AT2*^Micu1^^KO^* mice compared with *Sftpc^CreERT2^* mice (Figure 5, B and C). TUNEL staining of lung sections showed that compared with *Sftpc^CreERT2^* control lungs at 2 dpi, the number of apoptotic cells and apoptotic lineage-labeled AT2 (TUNEL^+^/GFP^+^) cells increased in AT2*^Micu1^^KO^* lungs (Figure 5D and Supplemental Figure 4E). The proliferation index was higher in AT2*^Micu1^^KO-EYFP^* lungs than that in the *Sftpc^CreERT2^*; *Rosa26^EYFP^* lungs at 4 dpi, as revealed by the percentage of lineage-labeled AT2 cells (GFP^+^) that were either EdU^+^ or Ki67^+^ (Figure 5E). These results indicate that loss of *Micu1* in AT2 cells did not affect bacterial load but led to increased cell apoptosis and AT2 cell proliferation after SpT4 infection–induced lung injury.

**Figure 6:**
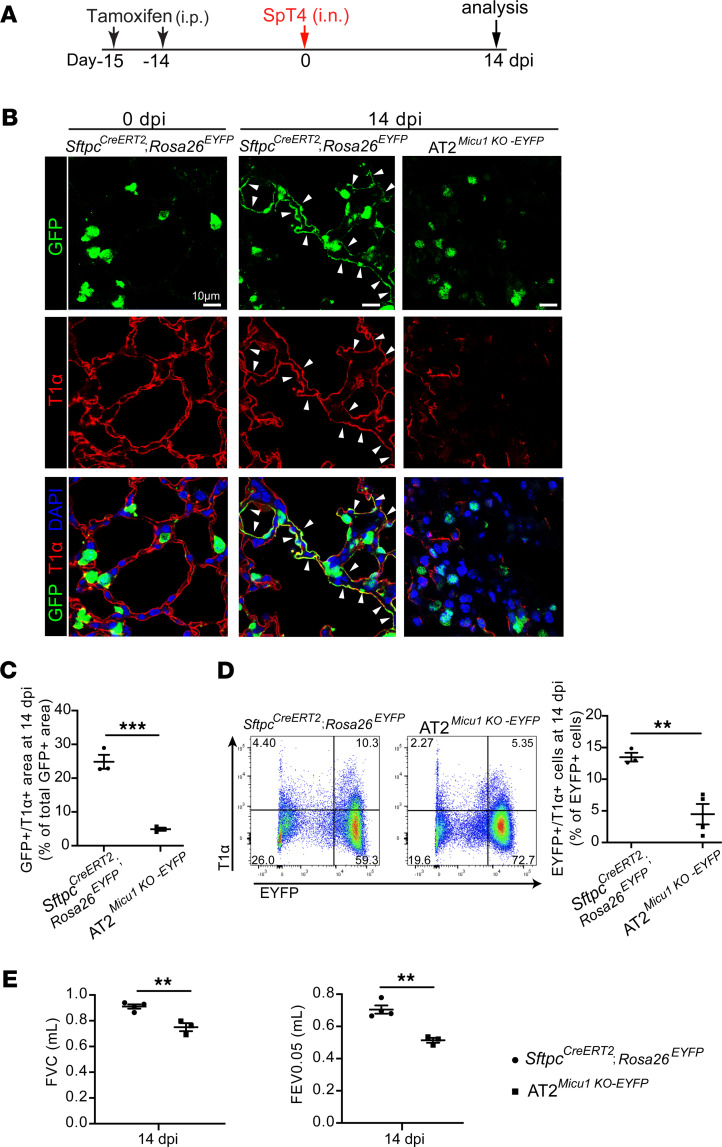
MICU1 expression is necessary for AT2-to-AT1 differentiation during alveolar epithelial repair and regeneration after SpT4 infection–induced injury. (**A**) Time line of tamoxifen treatment, SpT4 infection, and lung harvest from adult mice. (**B**) Confocal images of lung sections at 14 dpi with antibodies to GFP (green) and T1α (red). GFP antibody was used to detect EYFP+ cells. The cell nucleus was stained with DAPI (blue). Arrows point to regions double positive for GFP and T1α. Scale bar: 10 μm. (**C**) Quantification of the percentage of GFP+/T1α+ area of total GFP+ area per field using ImageJ. (**D**) Flow cytometry analysis of dissociated lung cells showing the percentage of EYFP+/T1α+ cells from the total of EYFP+ cells at 14 dpi. (**E**) Pulmonary functions (namely, FVC and FEV_0.05_) measured at 14 dpi. Data are presented as mean ± SEM. *P* values were calculated using Student’s *t* test. **P < 0.01; ***P < 0.001.

We next investigated the effect of loss of *Micu1* on the differentiation of AT2 cells into AT1 cells. Notably, AT2-to-AT1 cell differentiation was significantly reduced in AT2*^Micu1^^KO-EYFP^* mice during alveolar repair and regeneration (Figure 6, A–D). At 14 dpi, flow cytometry analysis revealed that 13.5% ± 1.9% of total lineage-labeled GFP^+^ cells in control lungs were T1α^+^ (EYFP^+^/T1α^+^) (Figure 6D). In contrast, EYFP^+^/T1α^+^ cells were reduced by 66.7% (4.5% ± 1.9%) in AT2*^Micu1^^KO-EYFP^* lungs (Figure 6D), indicating decreased AT2-to-AT1 cell differentiation. Analysis of lung mechanics by flexiVent revealed significant decreases in FVC and FEV_0.05_ in AT2*^Micu1^^KO-EYFP^* mice (Figure 6E). Collectively, these data indicate that loss of *Micu1* in AT2 cells inhibited AT2-to-AT1 cell differentiation, resulting in decreased pulmonary function and slow recovery of surviving animals from bacterial pneumonia.

## Discussion

Despite AT2 cell plasticity becoming a very intense field of research, the molecular mechanisms regulating the differentiation process are still emerging. Advances in imaging technology have provided rich information about the ultrastructure of lung epithelial lineage progenitor cells, including provocative observations regarding the unique organelle structure of AT2 cells. However, this body of information, to a large extent, is descriptive, and the actual functions of some of the organelles, including mitochondria, in the biology of AT2 cells have remained unclear. _m_Ca^2+^ modulates bioenergetics, which is essential for multiple cellular activities, including cell proliferation, differentiation, survival, and the inflammatory response. _m_Ca^2+^ uptake via the mtCU is regulated by MICU1 (5–7, 13–15, 20, 21). In this work, we demonstrated in two ways that _m_Ca^2+^ uptake regulates AT2-to-AT1 cell differentiation. First, our in vitro culture model demonstrated that the MICU1 expression increases during AT2-to-AT1 cell differentiation. Increased MICU1 and increased MICU1-to-MCU ratio are required to decrease _m_Ca^2+^ uptake in the context of AT2-to-AT1 cell differentiation. Second, in vivo functional analysis showed that MICU1 expression is necessary for AT2-to-AT1 cell differentiation during both homeostatic lung maintenance and epithelial regeneration after SpT4 infection–induced lung injury.

Using a combination of cellular and genomic approaches, we have established a direct and casual role for MICU1 in efficient alveolar epithelial repair and regeneration in response to SpT4-induced lung injury. We demonstrate that _m_Ca^2+^ uptake is necessary for AT2 cell differentiation into AT1 cells. Our data provide strong experimental evidence for the hypothesis that MICU1-gated mtCU and their corresponding _m_Ca^2+^ uptake are essential for the expression of differentiation genes in mammalian AT2 cells and functionally important for the fate transition of AT2 cells to the AT1 cell lineage. A recent profiling of mtCU components in human embryonic stem cells and induced pluripotent stem cells suggests that MICU1 expression may set the stage for cellular differentiation and maturation (26). Our results are consistent with these findings and also provide strong support for the functional significance of MICU1 expression in a mammalian lung system.

A remaining question is what role MICU1-dependent _m_Ca^2+^ uptake plays in either the initiation or maintenance of AT2-to-AT1 cell differentiation. The mitochondrial metabolism hypothesis postulates that _m_Ca^2+^-dependent metabolic reprogramming functions in a combinatorial way to regulate events such as histone modifications and transcriptional activation (42). Thus, MICU1-dependent _m_Ca^2+^ uptake could directly affect TCA cycle metabolite bioavailability, resulting in the activation of epigenetic modifiers for changes in chromatin structure. In support of this model, reduced _m_Ca^2+^ uptake increased demethylation of histone H3K27 by JMJD3 via altering α-ketoglutarate bioavailability (28, 43). Additionally, in mouse embryonic fibroblasts, ablation of _m_Ca^2+^ uptake promotes the conversion of quiescent fibroblasts into myofibroblasts in an α-ketoglutarate–dependent fashion (28). Our analysis indicates that, in mouse adult AT2 cells, sustained _m_Ca^2+^ uptake reduces AT2 cell differentiation into AT1 cells.

Our results also reveal that _m_Ca^2+^ uptake regulates AT2 cell survival and proliferation. MICU1 expression is necessary for AT2 cell survival after epithelial injury, and loss of MICU1 led to excessive _m_Ca^2+^ uptake, which could contribute to exacerbated epithelial injury–induced cell death. In addition, loss of MICU1 in AT2 cells increased AT2 cell proliferation. These results support the finding that _m_Ca^2+^ uptake regulates cell cycle progression and proliferation in skin fibroblasts during wound healing (44).

Our study is not without limitations. We show that sustained elevation of miR-302 in adult AT2 cells decreases AT2-to-AT1 cell differentiation. Although our studies show that miR-302 targets and inhibits MICU1 expression, it is possible that other downstream targets of miR-302 operate in parallel to influence AT2-to-AT1 cell differentiation. Another limitation was the use of C57BL/6J mice. This substrain contains a mutation in the nicotinamide nucleotide transhydrogenase, which may affect the baseline mitochondrial redox balance (45). At this stage, we have no way to determine whether these altered mitochondrial redox balances could affect some of our mitochondrial readouts. However, we believe that our reported changes in _m_Ca^2+^ transients need to be considered estimates rather than accurate numeric estimations. Although MICU1-dependent _m_Ca^2+^ uptake is important for AT2 cell plasticity in our mouse model of bacterial pneumonia, it is not possible from these experiments to extrapolate our findings to all species or all models of lung injury. Additional studies are needed to assess whether regulation of AT2 cell plasticity by MICU1-dependent _m_Ca^2+^ uptake can be implicated in chronic lung diseases as well.

In conclusion, we have demonstrated that MICU1-dependent _m_Ca^2+^ uptake plays an essential role in regulating AT2 cell differentiation into AT1 cells during steady-state tissue maintenance and alveolar epithelial regeneration after SpT4 infection–induced lung injury. Future investigations will focus on how the _m_Ca^2+^ uptake regulates the initial patterns and maintenance of AT2-to-AT1 cell differentiation gene expression.

## Methods

### Mice.

The following mouse strains were used in this study: C57BL/6 (The Jackson Laboratory), *Sftpc^CreERT2^* (31), *Rosa26^miR-302^* (32), *Rosa26^mTmG^* (The Jackson Laboratory), *Rosa26^EYFP^* (The Jackson Laboratory), *Yap^fl/fl^* (31), *Taz^fl/fl^* (31), and *Micu1^fl/fl^* (25). Both sexes of mice were used and kept on a mixed genetic background.

### Tamoxifen and EdU administration.

Tamoxifen (catalog T5648; Sigma) was dissolved in corn oil (catalog C8267; MilliporeSigma) to make a 20 mg/mL stock solution. Mice were given via i.p. injection (200 mg/kg) to activate the Cre recombinase. EdU was administered via i.p. injection (50 mg/kg), followed by a chase of 3 hours.

### Antibodies.

The following antibodies were used for immunostaining: hamster monoclonal (8.1.1) anti-mouse T1α (1:200; Developmental Studies Hybridoma Bank, University of Iowa); goat polyclonal anti-GFP (1:200; Abcam, ab6673); rabbit monoclonal (SP6) anti-mouse Ki67 (1:100; Abcam, ab16667); chicken anti-goat IgG (H+L) secondary antibody, Alexa Fluor 488 (1:500; Thermo Fisher Scientific, A-21467); goat anti-rabbit IgG (H+L) secondary antibody, Alexa Fluor 568 (1:500; Thermo Fisher Scientific, A-11011); and goat anti-hamster IgG (H+L) secondary antibody, Alexa Fluor 568 (1:500; Thermo Fisher Scientific, A-21112). The following antibodies were used for flow cytometry: rat monoclonal anti-mouse CD45 APC/Cyanine7 (1:100; Biolegend, 103116); hamster monoclonal (clone 8.1.1) anti-mouse T1α-Brilliant Violet 421 (1:300; Biolegend, 127423); and rat monoclonal anti-mouse epithelial cell adhesion molecule (Ep-CAM) (CD326) Brilliant Violet 605 (1:100; Biolegend, 118227). The following antibodies were used for Western blotting: rabbit monoclonal (D4P8Q) anti-mouse MICU1 (1:1000; Cell Signaling Technology, 12524); rabbit monoclonal (D2Z3B) anti-mouse MCU (1:1000; Cell Signaling Technology, 14997); rabbit polyclonal anti-mouse MICU2 (1:1000; Abclonal, A12198); rabbit polyclonal anti-mouse MCUR1 (1:1000; Cell Signaling Technology, 13706); rabbit polyclonal anti-mouse SP-A (1:1000; Abclonal, A3133); rabbit polyclonal anti-mouse SP-B (1:1000; Abclonal, A1748); rabbit polyclonal anti-mouse SP-D (1:1000; Abclonal, A1651); rabbit monoclonal (D8T4N) anti-mouse Tom20 (1:1000; Cell Signaling Technology, 42406); mouse monoclonal (E-1) anti-HOPX (1:1000; Santa Cruz, sc-398703); and mouse monoclonal (C4) anti-gizzard actin of chicken origin β-actin (1:1000; Santa Cruz, sc-47778). TUNEL staining was performed using In Situ Cell Death Detection Kit (Roche). EdU incorporation assay was performed using Click-iT EdU Alexa Fluor Imaging Kit (Thermo Fisher Scientific).

### In vitro 2D culture model.

Primary AT2 cells from mice 6–10 weeks old were isolated as described previously (2, 36). Purified primary AT2 cells in complete mouse medium (DMEM/F12; 1 mM l-glutamine; 0.25% BSA; 10 mM HEPES; 0.1 mM nonessential amino acids; 0.05% insulin-transferrin-sodium selenite; 100 U/mL penicillin G; 100 μg/mL streptomycin) plus 2% FBS were cultured on plastic dishes coated with rat-tail collagen (prepared in our laboratory). Cell culture medium was replaced with serum-free complete mouse medium 2 days after plating and every other day thereafter.

### Live-cell imaging of calcium transients.

Primary mouse AT2 cells were cultured for 24 hours in undifferentiation medium (complete mouse medium plus 2% FBS) or for 7 days in differentiation medium (serum-free complete mouse medium). Prior to live-cell imaging, AT2 cells were washed with PBS and loaded with Ca^2+^-sensitive dye X-Rhod-1 (1 μM; Invitrogen) or Fluo-4 AM (1 μM; Invitrogen) for 30 minutes to measure mitochondrial or cytosolic Ca^2+^ transients, respectively. Cells were washed with PBS and placed in a 37°C heated chamber in phenol-red–free, serum-free complete mouse medium and imaged on a ZEISS LSM 900 with Airyscan 2 microscope. Ca^2+^ transients were continuously recorded and analyzed with Zen Blue software (ZEISS). After 2 minutes of baseline recording, a single pulse of 1 mM ATP was delivered to liberate intracellular Ca^2+^ stores. Background fluorescence was subtracted from each experiment before calculating the peak intensity as the maximal fluorescence divided by baseline fluorescence. For cell permeabilization assay, cells were loaded with MitoTracker Green (1 μM; Invitrogen) and X-Rhod-1, permeabilized with 20 μg/mL digitonin, and imaged before and after digitonin treatment.

### Bacterial infection.

The pneumococcal strain used was the clinically isolated SpT4 (43). SpT4 was stored at –80°C and grown in tryptic soy agar plus catalase (57 μgP/mL) under microaerophilic conditions for 14–16 hours at 37°C with 5% CO_2_, then subcultured and grown to an OD of 0.5. The broth was centrifuged, and the bacteria were washed in sterile PBS and resuspended in sterile PBS immediately prior to infection. Mice were anesthetized at 8–10 weeks of age using a ketamine plus xylazine mixture and infected intranasally with a dose of approximately 5 × 10^6^ CFU in 30 μL of sterile PBS.

### Bacterial load measurement.

Pneumococcal loads were determined by homogenization of lung tissue in sterile PBS at 2 days after infection of the animals. Tissue homogenate (100 μL) and additional dilutions were plated on tryptic soy agar plates plus catalase (57 μgP/mL) for culture overnight at 37°C with 5% CO_2_, and the number of CFU was counted.

### Mouse lung dissociation and flow cytometry.

Lungs were dissociated using previously described protocols (31). Lungs were instilled with dispase (25 U/mL, 37°C) through the trachea and were incubated in dispase for digestion for 6 minutes at 37°C. Each lung lobe was then minced in DMEM containing DNase I (120 U/mL) followed by a rotating incubation for 10 minutes at room temperature. Cells were then filtered sequentially through 100 and 40 μm strainers on ice and centrifuged at 200*g* for 10 minutes at 4°C. Cells were incubated with RBC lysis buffer for 1 minute on ice and centrifuged, and then washed with PBS. Cells were resuspended in FACS buffer (1× PBS, 1% BSA, 0.1% NaN_3_). Dissociated cells were blocked with anti-mouse CD16/32 (catalog 101320; Biolegend) at 1:100 for 20 minutes. Cells were then incubated with fluorophore-conjugated antibodies for 1 hour. For live cell staining, LIVE/DEAD Fixable Aqua Dead Cell Stain (catalog L34966A; Thermo Fisher Scientific) was added along with fluorophore-conjugated antibody mix at a 1:1600 dilution. Cells were washed twice with FACS buffer and resuspended in FACS buffer. Epithelial cells were selected using the CD45-negative fraction of the cell isolate that stained positively for Ep-CAM (CD326). Within the epithelial cell gate, EYFP^+^, T1α^+^, or EYFP^+^/T1α^+^ cells were identified and quantified by their geometric mean fluorescence signal intensity. Data were acquired using a BD LSR II flow cytometer and analyzed using FlowJo, version 10.4.2 (BD).

### Cell lines, transfection, and LUC reporter assay.

HEK293T cells were purchased from American Type Culture Collection and grown in DMEM containing 10% FBS. DNA transfections were done with X-tremeGENE HP DNA transfection reagent (Roche) in antibiotic-free medium according to manufacturer’s instructions. The firefly LUC constructs were generated as described previously (30). Briefly, the DNA fragment containing full-length 3′-UTR of *Micu1* was inserted into pcDNA3.1(–) downstream of the LUC cDNA. Cells were transfected with both the micu1 3′-UTR reporter (pcDNA3.1-luc-*Micu1* 3′-UTR) and an expression plasmid for miR-302-367 (pcDNA3.1-miR-302-367). The Ctrl group was cells transfected with empty LUC reporter (pcDNA3.1-luc) and pcDNA3.1-miR-302-367. Cell extracts were assayed for LUC expression at 48 hours after transfection using a commercially available kit (Promega). The following primers are used for LUC reporter assay cloning (lowercase letters indicate enzyme sites for cloning): Micu1 XhoI forward: 5′-agccctcgagTATTCCCACCTCCTGCACC-3′, Micu1 KpnI reverse: 5′-agccggtaccTTGCGGGTATTTCCTGCAAAG-3′; Micu1 site mutagenesis forward: 5′-AATATCACTTTAATTATAGTACTATGCAGGAAATACCCGC-3′, Micu1 site mutagenesis reverse: 5′-GCGGGTATTTCCTGCATAGTACTATAATTAAAGTGATATT-3′. Relative reporter activities are expressed as luminescence units normalized for β-galactosidase expression in the cell extracts.

### Gene expression analysis.

qPCR analysis was performed as described before (31). The following primers are used for qPCR analysis: Micu1: forward: 5′-TGTGGGCTCATCTCCTTCTC-3′, reverse: 5′-CCTCTCCGTCTCCATTCAAGT-3′; *Mcu:* forward: 5′-ATGGCCATGTATGCGTATTTTG-3′, reverse: 5′-TCGAAACGTGACTTTTTGGC-3′; *Emre,* forward: 5′-TTTTGTCCCAGAGGATGACG, reverse: 5′-CATGCCACCACATCATCAGT-3′; *Mcur1:* forward: 5′-GAACTTGCCCCTCTCTGTGA-3′, reverse: 5′-TCTTGACCAGTGCAGACACAA-3′; *Rps13:* forward: 5′-ACGTCTGACGACGTGAAGGAAC-3′, reverse: 5′-TTTCCAGTCACAAAACGGACCTG-3′.

### Western blot.

Whole-cell proteins were extracted as described previously, with minor modifications (31). Briefly, cells were lysed using RIPA buffer containing protease and phosphatase inhibitors. For extraction of proteins from tissues, lungs were homogenized in RIPA buffer, using a tissue grinder. Protein concentrations were determined using the BCA Protein Assay Reagent kit (Bio-Rad Laboratories). Protein extracts were analyzed on polyacrylamide gels and transferred to nitrocellulose membrane. The blots were blocked in 5% milk in PBST at room temperature for 1 hour, followed by incubation with primary antibody diluted in blocking buffer at room temperature for 1 hour. The blots were washed five times with PBST and incubated with HRP-conjugated secondary antibodies diluted in blocking buffer at room temperature for 1 hour, then developed with Thermo Scientific SuperSignal West Femto Maximum Sensitivity ECL substrate and imaged with GE Healthcare ImageQuant LAS 4000.

### Tissue harvest and immunostaining for histology and quantification.

Lungs were inflated using the gravity drip as previously described (31). Mice were euthanized by Avertin overdose (300 mg/kg) followed by cervical dislocation. Lungs were perfused with 10 mL of PBS through the right ventricle. Lungs were inflated with 4% paraformaldehyde (PFA) at 25 cm H_2_O pressure gravity drip. For transmission electron microscopy analysis, lungs were inflated with 2% glutaraldehyde and 2% PFA. The trachea was then tied off and intact lungs were immersed in 4% PFA for 4 hours at 4°C. Lung lobes were separated and washed with cold PBS overnight. After ethanol dehydration, lungs were embedded in paraffin and sectioned at 6 μm. To perform immunohistochemical staining, slides were deparaffinized and rehydrated. Tissue sections were then incubated in citrate buffer (pH 6.0) for 20 minutes at 95°C to 100°C, followed by permeabilization and blocking with 0.3% Triton X-100 and 5% goat serum or horse serum (for goat primary antibodies) in PBS for 1 hour at room temperature. Primary antibodies were diluted in PBS and incubated overnight at 4°C. Secondary antibodies were diluted in PBS and incubated for 1 hour at room temperature. EdU staining, TUNEL staining, and DAPI nuclear staining were performed according to manufacturer recommendations. Tissue sections were washed 3 times with PBS between antibody incubations for 15 minutes each. Slides were mounted with Aqua-Poly/Mount and images were captured on a Zeiss LSM 710 confocal microscope and a Nikon eclipse fluorescence microscope. Images were processed with ImageJ/FIJI. Quantitation of cell numbers was completed using at least 10 randomly selected regions of each lung per animal. Mitochondrial size and cristae numbers (Figure 2E) were calculated by measuring the surface area of each mitochondrion and outlining each crista within the mitochondrion using NIH ImageJ (46).

### Lung lavage fluid and blood collection and phosphatidylcholine measurements.

Avertin was used to anesthetize mice. Bronchoalveolar lavage was performed with 800 μL lavages of sterile saline using a 20G blunt-tipped needle inserted into the trachea. Samples were centrifuged at 400*g* for 10 minutes at 4°C, and the supernatant (BALF) was transferred to a clean tube. Phosphatidylcholine in the BALF was measured using a phosphatidylcholine colorimetric assay kit (catalog 10009926; Cayman) following the manufacturer’s instructions.

### Statistics.

Data are reported as mean ± SEM. Multiple groups were compared by 1-way ANOVA followed Tukey’s or Dunnett’s post hoc test. Two-tailed Student’s *t* test was used when comparing 2 experimental groups. Multiple groups with multiple time points were compared by 2-way ANOVA followed by Šidák multiple comparisons test. *P* < 0.05 was considered significant. All analyses were performed with GraphPad Prism 9.

### Study approval.

This study was conducted according to the guidelines outlined by the Public Health Service Policy on the Human Care and Use of Laboratory Animals. All protocols for breeding and experiments with animals were approved by the Temple University IACUC (protocol no. 5012).

## Author contributions

MA and XZ performed most of the experiments. RL performed immunostaining and quantification of AT2 cells in *Sftpc^CreERT2^*; *Rosa26^miR-302^*; *Rosa26^mTmG^* lungs. DT and JWE advised on calcium imaging. YT supervised all experiments and wrote the manuscript with feedback from the coauthors. All authors read, edited, and approved the manuscript.

## Supplementary Material

Supplemental data

## Version 1 01/20/2022

In-Press Preview

## Version 2 02/22/2022

Electronic publication
