# Supplementary material for: MICU1-dependent mitochondrial calcium uptake regulates lung alveolar type 2 cell plasticity and lung regeneration
Source: JCI Insight. 2022 Feb 22;7(4):e154447. doi: 10.1172/jci.insight.154447 (PMC8876408; doi:10.1172/jci.insight.154447)
Supplement: Supplemental data [file jciinsight-7-154447-s229.pdf]

Supplemental Figure 1

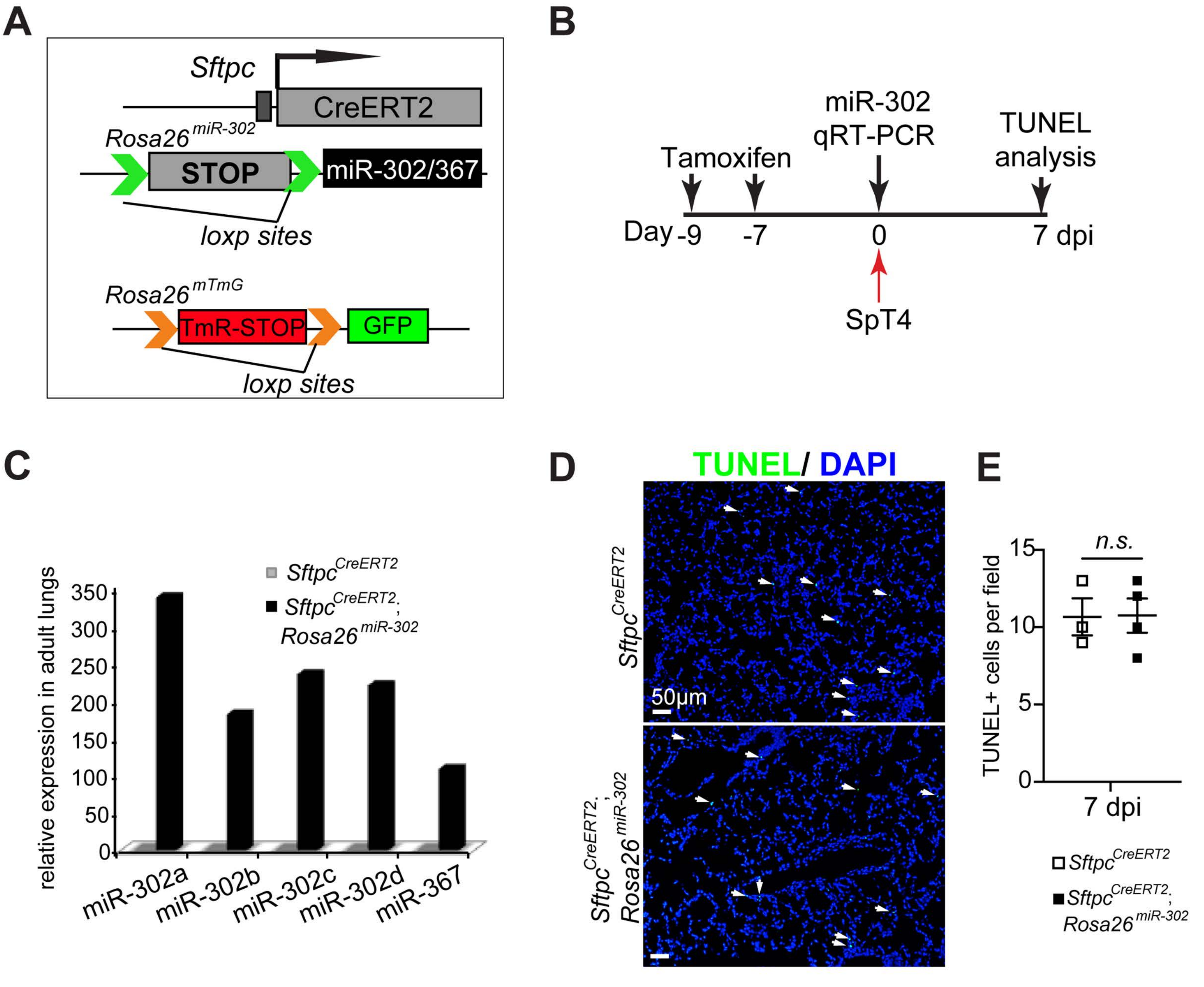

Supplemental Figure 1. Overexpression of miR-302 in adult AT2 cells. (A) Schematic of experimental design for generating *Sftpc*^{CreERT2}; *Rosa26*$^{miR\text{-}302}$; *Rosa26*^{mTmG} mice. (B) Adult *Sftpc*^{CreERT2} or *Sftpc*^{CreERT2}; *Rosa26*$^{miR\text{-}302}$ mice were administrated with two doses of tamoxifen. After 7 days of last tamoxifen treatment, mice were either processed for AT2 cell purification or infected with SpT4. For SpT4-infected mice, lungs were examined at 7 days post infection (dpi). (C) Quantification of expression of all members of miR-302 cluster in purified AT2 cells by qPCR at 0 dpi. (D) TUNEL staining of lung sections at 7 dpi. Arrows point to TUNEL+ cells. Scale bars: 50 µm. (E) Quantification of cell apoptosis by counting TUNEL+ cells on lung sections. Data are presented as mean ± s.e.m. P values were calculated using Student's t test.

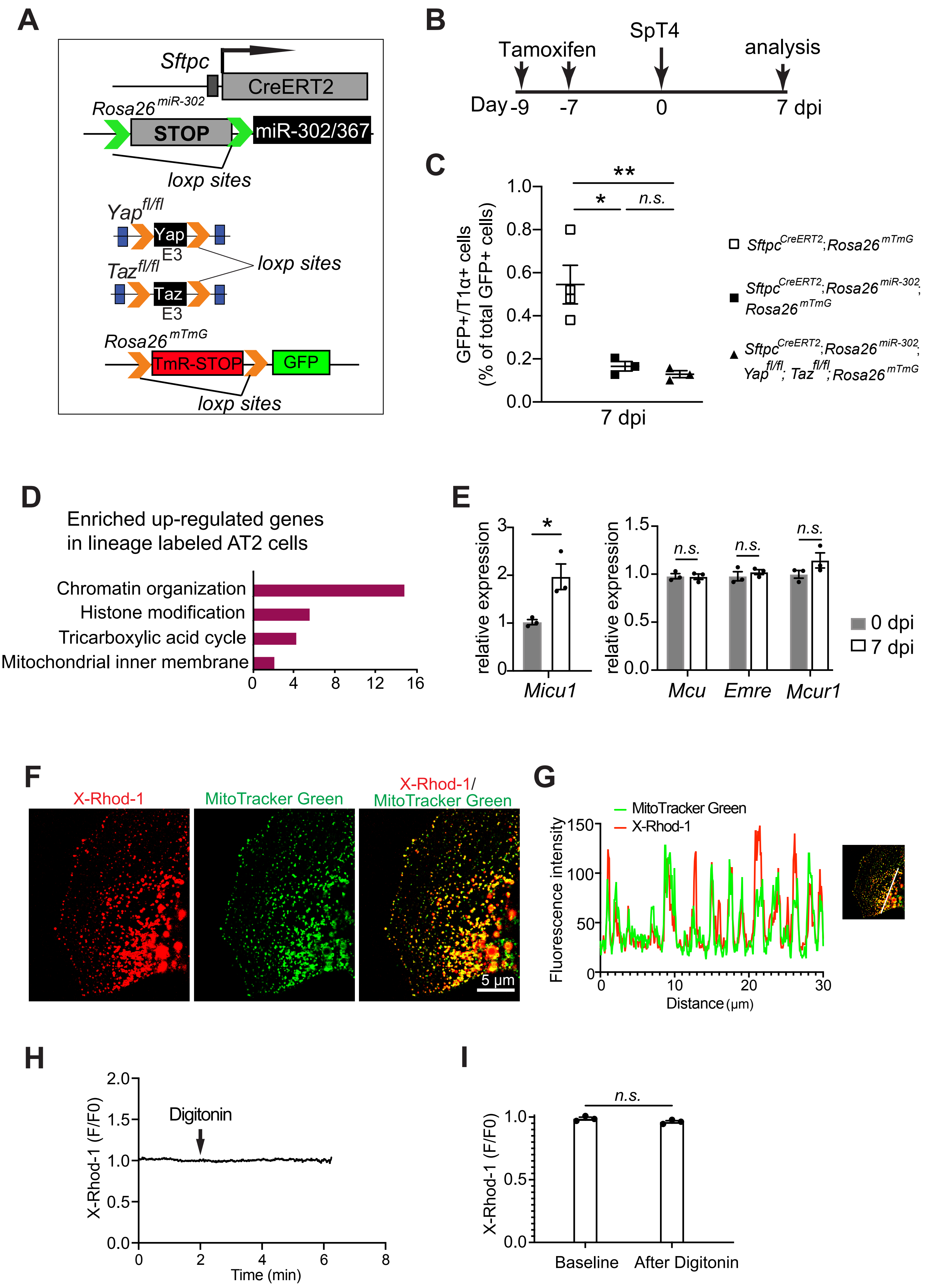
A
Sftpc
CreERT2
Rosa26 miR-302
STOP
miR-302/367
loxp sites
Yap fl/fl
Yap
E3
Taz fl/fl
Taz
E3
loxp sites
Rosa26 mTmG
TmR-STOP
GFP
loxp sites
B
Tamoxifen
SpT4
analysis
Day -9
-7
0
7 dpi
C
GFP+/T1α+ cells (% of total GFP+ cells)
**
*
n.s.
7 dpi
Sftpc CreERT2;Rosa26 mTmG
Sftpc CreERT2;Rosa26 miR-302;Rosa26 mTmG
Sftpc CreERT2;Rosa26 miR-302;Yap fl/fl; Taz fl/fl;Rosa26 mTmG
D
Enriched up-regulated genes in lineage labeled AT2 cells
Chromatin organization
Histone modification
Tricarboxylic acid cycle
Mitochondrial inner membrane
E
relative expression
*
n.s.
n.s.
n.s.
Micu1
Mcu
Emre
Mcur1
0 dpi
7 dpi
F
X-Rhod-1
MitoTracker Green
X-Rhod-1/MitoTracker Green
5 μm
G
MitoTracker Green
X-Rhod-1
Fluorescence intensity
Distance (μm)
H
X-Rhod-1 (F/F0)
Digitonin
Time (min)
I
n.s.
X-Rhod-1 (F/F0)
Baseline
After Digitonin

Supplemental Figure 2. The miR-302 reduction of AT2 cell differentiation into AT1 cells is maintained in Yap/Taz knockout mice. (A) Schematic of experimental design for generating *Sftpc*^{CreERT2}; *Rosa26*$^{miR\text{-}302}$; ***Yap***$^{fl/fl}$; *Taz*$^{fl/fl}$; *Rosa26*^{mTmG} mice. (B) Timeline of tamoxifen treatment, SpT4 infection, and lung harvest of adult mice for studies performed in (C and E). (C) Flow cytometry analysis on dissociated lung cells showing the percentage of GFP+/T1α+ cells of total GFP+ cells at 7 dpi. (D) Enriched Gene Oncology terms for genes up-regulated in lineage labeled AT2 cells from SpT4-infected lungs at 8 dpi compared with non-infected lungs of *Sftpc*^{CreERT2}; *Rosa26*^{mTmG} mice (GSE86027). Gene Oncology associations and related *P* values were determined using ToppGene Suite (https://toppgene.cchmc.org). (E) qPCR analysis showing gene expression in purified AT2 cells from SpT4-infected lungs (7 dpi) and non-infected lungs (0 dpi) of *Sftpc*^{CreERT2} mice. Gene expression was normalized to *Rps13*, a mitochondrial gene. (F) Confocal images of AT2 cells (day 9 of culture) loaded with X-Rhod-1 (red) and MitoTracker Green (green). (G) Intensity profiles of X-Rhod-1 (red) and MitoTracker green (green), along an ideal straight line crossing a representative cell (right inset). (H) X-Rhod-1 fluorescent trace from a single AT2 cell before permeabilization with digitonin (baseline) and after permeabilization with digitonin. (I) Quantification of X-Rhod-1 fluorescent trace at baseline and after permeabilization with digitonin. Data are presented as mean ± s.e.m. P values were calculated using one-way ANOVA (C) and Student’s t test (E, I).

Supplemental Figure 3

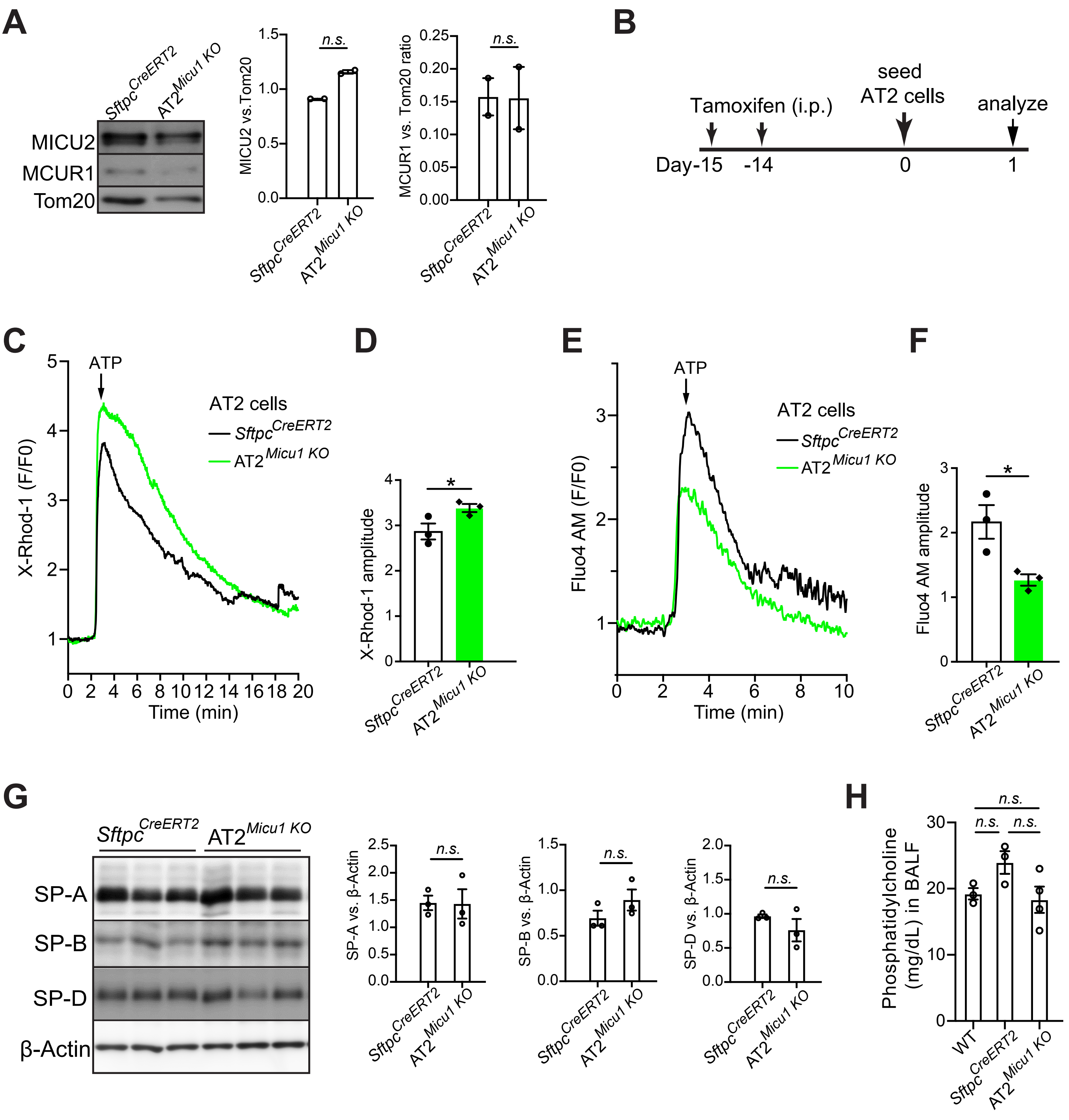

Supplemental Figure 3. Effects of MICU1 deletion on AT2 cell function during steady-state tissue maintenance. (A) Western blots of whole-cell protein from purified AT2 cells from *Sftpc*^{CreERT2} and *Sftpc*^{CreERT2}; *Micu1*$^{fl/fl}$ (AT2$^{Micu1\ KO}$) mice at 14 days after last dose of tamoxifen treatment. The fold change in the protein levels of MICU2 and MCUR1 by western blots was graphed on the right. Band density was normalized to Tom20. (B) Schematic of experimental design for studies performed in (C-F). (C) Measurement of $_{m}Ca^{2+}$ uptake in AT2 cells as assessed by the mitochondrial calcium sensor, X-Rhod-1. 1 mM ATP was delivered to initiate IP3R-mediated Ca^{2+} release. (D) Amplitude (peak intensity) of X-Rhod-1 was graphed. (E) Measurement of $_{c}Ca^{2+}$ uptake in AT2 cells as assessed by the cytosolic calcium sensitive dye Fluo4 AM. (F) Amplitude (peak intensity) of Fluo4 AM was graphed. (G) Western blots of whole-cell protein from lung tissues. The fold change in the protein levels of SP-A, SP-B, and SP-D by western blots was graphed on the right. Band density was normalized to β-Actin. (H) Measurement of phosphatidylcholine in bronchoalveolar lavage fluid (BALF) using phosphatidylcholine colorimetric assay. Data are presented as mean ± s.e.m. P values were calculated using Student's t test (A, D, F, G) and one-way ANOVA (H).

Supplemental Figure 4

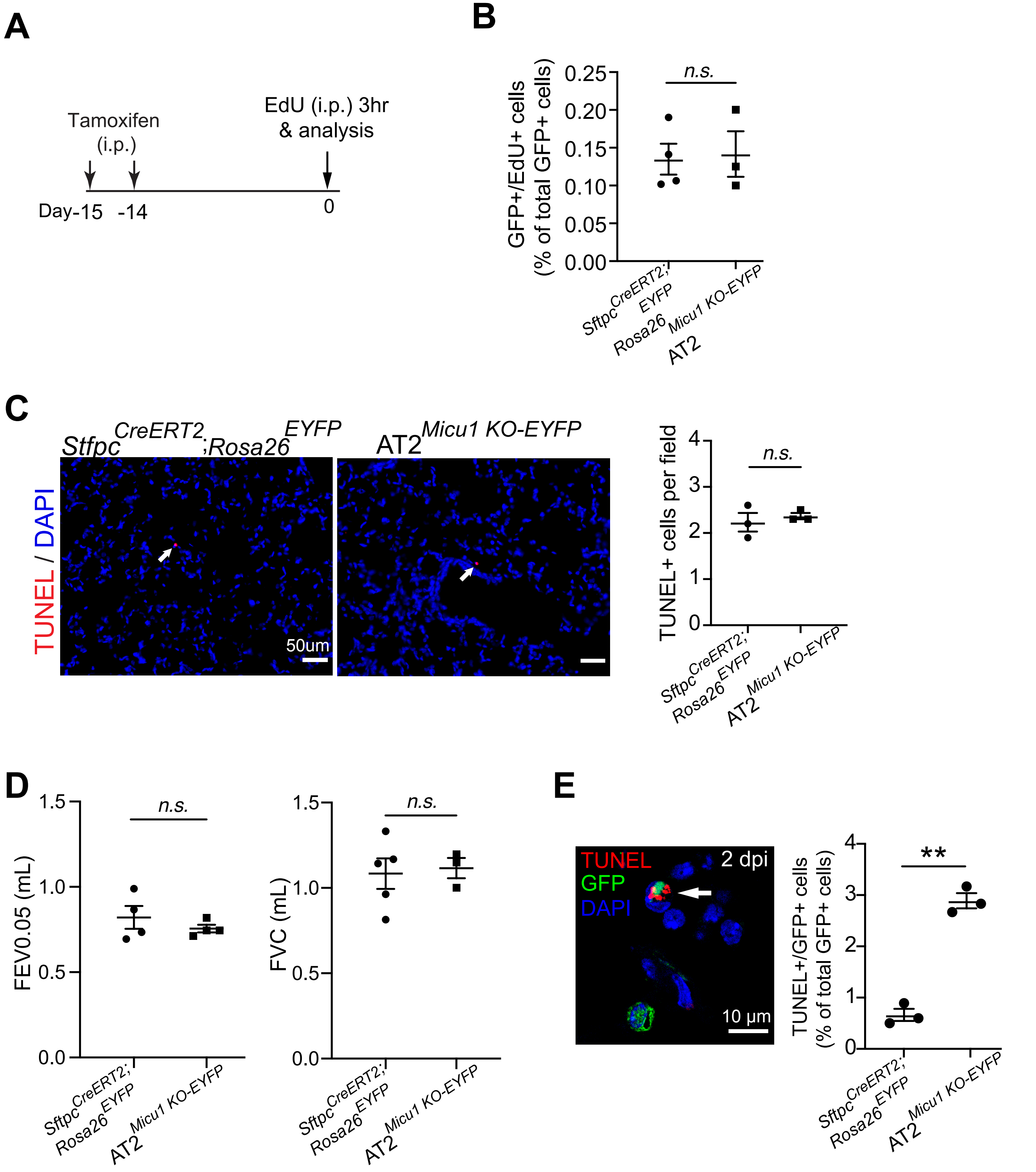

Supplemental Figure 4. Effects of MICU1 deletion on AT2 cell proliferation, cell apoptosis and pulmonary function at homeostasis. (A) Schematic of experimental design for studies performed in (B-D). Adult *Sftpc*^{CreERT2}; *Rosa26*^{EYFP} (Ctrl) and *Sftpc*^{CreERT2}; *Micu1*$^{fl/fl}$; *Rosa26*^{EYFP} (AT2$^{Micu1\ KO\text{-}EYFP}$) mice were administrated two doses of tamoxifen and examined at 14 days after last tamoxifen treatment. (B) Quantification of EdU+/GFP+ cells as percentage of total GFP+ cells analyzed (~1,000 GFP+ cells per animal) on lung sections. (C) TUNEL staining of lung sections. Graph on the right showing the quantification of cell apoptosis by counting TUNEL+ cells on lung sections. (D) Pulmonary functions were analyzed for forced expiratory volume in 0.05 seconds (FEV0.05) and forced vital capacity (FVC). (E) Confocal images of TUNEL (red)-stained lung sections at 2 dpi co-immunostained with GFP (green) antibody. GFP antibody was used to detect EYFP+ cells (lineage-labeled AT2 cells). Cell nuclear was stained with DAPI (blue). Arrows point to regions double positive for GFP and TUNEL. Scale bars: 10 μm. Graph on the right showing quantification of TUNEL+/GFP+ cells as percentage of total GFP+ cells analyzed on lung sections. Data are presented as mean ± s.e.m. P values were calculated using Student's t test.
